# Toward designer magic bullets: Epigenetic editing to empower CAR T cells

**DOI:** 10.1016/j.omtn.2025.102700

**Published:** 2025-09-13

**Authors:** Laura Horvathova, Valerie R. Wiersma, Marianne G. Rots

**Affiliations:** 1 Department of Pathology and Medical Biology, University of Groningen, University Medical Center Groningen, Groningen, the Netherlands; 2 Department of Hematology, University of Groningen, University Medical Center Groningen, Groningen, the Netherlands

## Main text

In this issue, Azcona et al. demonstrate the promise of “hit-and-run” epigenetic editing as a versatile approach to improve chimeric antigen receptor (CAR) T cell functionality.^1^ As proof of concept, the authors epigenetically repressed the expression of two key exhaustion-related genes in primary human T cells and prostate stem cell antigen-specific CAR T cells. The precise and durable gene silencing was induced by gene-targeted epigenetic reprogramming, which does not alter the DNA sequence, thereby offering a safe and reversible strategy to regulate CAR T cell function.

Despite their success in hematological malignancies, CAR T cell therapies are limited by antigen escape, as well as by immunosuppressive tumor microenvironments, and the challenges of effective homing and infiltration in solid tumors. Moreover, even in hematological malignancies, exhaustion features are observed in patients with low CAR T cell responsiveness.^2^ To overcome these issues and enhance CAR T cell efficacy, gene editing, primarily via CRISPR-Cas9-mediated gene knockout, is being actively pursued. For instance, preclinical studies across diverse cancer models have shown that CRISPR-Cas9-mediated disruption of the exhaustion marker programmed cell death 1 (*PDCD1*, encoding PD-1) enhances CAR T cell functionality and tumor clearance.^2^ Importantly, a phase 1 clinical trial demonstrated that PD-1 knockout MUC1-targeted CAR T cells were well tolerated in patients with breast cancer.^3^ Lymphocyte activation gene-3 (*LAG-3*) is another critical exhaustion marker, and CRISPR-mediated *LAG-3* knockout CAR T cells exhibited potent, antigen-specific cytotoxicity both *in vitro* and in murine models.^2^ Of note, dual targeting of *PD-1* and *LAG-3* had a synergistic effect, reversing CD8^+^ T cell exhaustion and boosting antitumor responses.^4^

Although these studies underscore the therapeutic potential of genetically modulating exhaustion pathways, the clinical use of CRISPR-Cas9 technology is associated with safety concerns as it exploits error-prone repair pathways and introduces permanent DNA changes that may be genotoxic. Epigenetic editing (the gene-targeted rewriting of epigenetic marks) circumvents these issues while being equally effective^5^^,^^6^ and potentially reversible.^6^

In their current study, Azcona et al. addressed CAR T cell exhaustion by simultaneously targeting *PDCD1* and *LAG-3*. They engineered epigenetic editors referred to as designer epigenome modifiers (DEMs). These DEMs consist of a transcription activator-like effector (TALE)-based programmable DNA-binding domain fused to either Krüppel-associated box (KRAB), a transcriptional repressor that indirectly induces repressive posttranslational histone modifications (H3K9me3), or a DNMT3A-DNMT3L fusion, which establishes repressive DNA methylation. Of note, by targeting both KRAB and DNMT3A/3L to genes of interest, the authors were among the first to demonstrate durable gene silencing by epigenetic editing.^7^ The epigenetic silencing of *PCDC1* and *LAG-3* by DEMs did not affect the expansion and viability of edited T cells compared to unmodified controls. The authors further reported that targeting these genes resulted in an approximately 2-fold decrease in PD-1^+^ and LAG-3^+^ cells, which persisted over multiple rounds of cell division and reactivation.

Intriguingly, the DEMs were introduced into target cells *via* transient mRNA electroporation, confirming the power of epigenetic editing as a hit-and-run approach. This non-viral strategy may thus offer a superior alternative to a previous multiplexing study using KRAB-based artificial transcription factors to silence immune checkpoints and TGFBR2 in CAR T cells and tumor-infiltrating lymphocytes.^8^ As KRAB alone generally induces transient effects, it requires the use of potentially hazardous lentiviral vectors to sustain gene silencing.

While CRISPR-dCas9, comprising the catalytically inactive form of Cas9, currently is the most widely used platform for epigenetic editing, Azcona et al. opted for TALEs. Unlike CRISPR, which exploits straightforward guide RNA engineering for gene targeting, TALEs (and zinc finger proteins) require custom protein engineering to bind a given target gene, making them more labor-intensive and less amenable to high-throughput applications. Nevertheless, their smaller sizes, target flexibility, lower immunogenicity and their protein-DNA interaction, which avoids the opening of the two DNA strands, offer an attractive alternative platform with proven clinical safety. Importantly, transcriptome-wide RNA sequencing analysis in the current study revealed minimal off-target effects, indicating the high specificity of the TALE-based DEM-mediated epigenetic editing.

A particularly impactful aspect of the study is the successful application of DEMs in PSMA-specific CAR T cells, which retained their proliferative capacity and expanded similarly to unmodified controls. In contrast, CAR T cells edited using multiplex CRISPR-Cas9 showed markedly reduced expansion, potentially induced by DNA damage responses. Moreover, the epigenetically silenced CAR T cells sustained the reduced frequency of PD-1^+^ and LAG-3^+^ cells even after antigen rechallenge. This suggests that repression is not only durable but also stable under conditions that partially mimic chronic antigen exposure in the tumor microenvironment.

Despite the strengths of the study, several questions remain. Notably, the epigenetically modified CAR T cells did not exhibit enhanced functional activity compared to the unmodified controls. This may be attributed to the use of a simplified model that lacked expression of the PD-1 and LAG-3 ligands, PD-L1, and major histocompatibility complex class II (MHC-II). In these settings, only modest cytotoxic improvements were observed at high effector-to-target ratios with T cells from one donor, but not another. Given that PD-1 blockade is known to be ineffective *in vitro*^8^ and in PD-L1-negative tumors,^9^ more pronounced effects might emerge in ligand-expressing systems. These could include models with interferon-γ-induced or genetically overexpressed PD-L1 and MHC-II, or ideally, autologous cocultures using matched patient-derived T cells and tumor cells that naturally express these ligand/receptor pairs. Furthermore, such models could clarify whether epigenetically edited CAR T cells perform on par with genetically edited counterparts. Lastly, the authors reported the proportions of PD-1^+^ and LAG-3^+^ cells, while acknowledging that these markers are not strictly binary and that their expression levels may have important functional implications. Eventually, fine-tuning expression (creating stable knockdowns, as achieved in this study by epigenetic editing) might indeed be more clinically favorable compared to inducing complete knock-outs as generated by gene editing.

**Figure 1:**
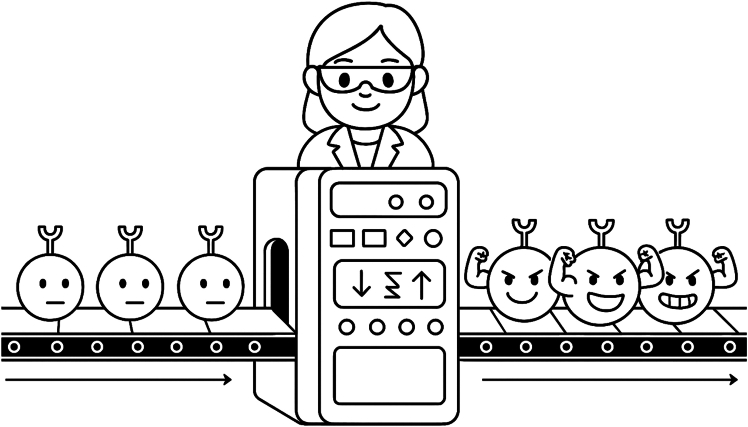
Reprogramming CAR T cells by tuning gene expression levels using epigenetic editing

Although the study focuses specifically on two exhaustion-related genes, it inspires a broader vision in which epigenetic editing could be harnessed to regulate a wide range of CAR T cell functions. By leveraging durability, precision, possibility for multiplexing and bidirectional expression modulation, while maintaining reversibility of epigenetic editing, future CAR T cell therapies could be engineered to fine-tune immune responses, minimize unwanted side effects, and overcome barriers such as exhaustion. In addition, the *ex vivo* manufacturing of CAR T cells (Figure 1) renders this therapy especially suited for editing platforms, as it bypasses *in vivo* delivery challenges and minimizes the risk of unintended effects in non-target cells. This strategy could furthermore be applied to the development of universal off-the-shelf CAR T cells by silencing T cell receptor and MHC genes, as proposed earlier.^10^ In this respect, the work of Azcona et al. offers both a technological blueprint, through non-viral mRNA delivery, and a conceptual framework, through multiplexed sustained knockdown, for implementing safe, flexible, and programmable editing in cellular immunotherapy. Reflecting its growing therapeutic promise, commercial interest, and the initiation of epigenetic editing clinical trials, underscores the momentum and translational potential of pursuing epigenetically edited CAR T cells.

Looking ahead, the field must now explore how best to deploy these tools, which genes to target, and the level of modulation needed. As epigenetic effectors continue to evolve, the ability to reversibly activate or repress multiple genes in parallel, with minimal off-target risks, brings an improved license to kill for next-generation, designer CAR T cells within reach.

## Declaration of interests

The authors declare no competing interests.

## Declaration of generative AI and AI-assisted technologies in the writing process

During the preparation of the Figure, the author(s) used ChatGPT version 5 in order to visualize the concept. After using this tool/service, the author(s) reviewed and edited the content as needed and take(s) full responsibility for the content of the publication.
